# Evidence for Involvement of Cytosolic Thioredoxin Peroxidase in the Excessive Resistance of Sf9 Lepidopteran Insect Cells against Radiation-Induced Apoptosis

**DOI:** 10.1371/journal.pone.0058261

**Published:** 2013-03-07

**Authors:** Shashank Hambarde, Vijaypal Singh, Sudhir Chandna

**Affiliations:** Natural Radiation Response Mechanisms Group, Division of Radiation Biosciences, Institute of Nuclear Medicine and Allied Sciences, Delhi, India; Wayne State University, United States of America

## Abstract

Lepidopteran insect cells display 50–100 times higher radioresistance compared to human cells, and reportedly have more efficient antioxidant system that can significantly reduce radiation-induced oxidative stress and cell death. However, the antioxidant mechanisms that contribute substantially to this excessive resistance still need to be understood thoroughly. In this study, we investigated the role of thioredoxin peroxidase (TPx) in high-dose γ-radiation response of Sf9 cell line derived from *Spodoptera frugiperda*, the Fall armyworm. We identified a TPx orthologue (Sf-TPx) in *Spodoptera* system, with primarily cytosolic localization. Gamma-irradiation at 500 Gy dose significantly up-regulated Sf-TPx, while higher doses (1000 Gy–2000 Gy) had no such effect. G2/M checkpoint induced following 500 Gy was associated with transition of Sf-TPx decamer into enzymatically active dimer. Same effect was observed during G2/M block induced by 5 nM okadaic acid or 10 µM CDK1 (cycline dependent kinase-1) inhibitor roscovitine, thus indicating that radiation-induced Sf-TPx activity is mediated by CDKs. Accumulation of TPx dimer form during G2/M checkpoint might favour higher peroxidase activity facilitating efficient survival at this dose. Confirming this, higher lethal doses (1000 Gy–2000 Gy) caused significantly less accumulation of dimer form and induced dose-dependent apoptosis. A ∼50% knock-down of Sf-TPx by siRNA caused remarkable increase in radiation-induced ROS as well as caspase-3 dependent radiation-induced apoptosis, clearly implying TPx role in the radioresistance of Sf9 cells. Quite importantly, our study demonstrates for the first time that thioredoxin peroxidase contributes significantly in the radioresistance of Lepidopteran Sf9 insect cells, especially in their exemplary resistance against radiation-induced apoptosis. This is an important insight into the antioxidant mechanisms existing in this highly stress-resistant model cell system.

## Introduction

Lepidopteran insects/insect cells, especially the moths and cell lines derived from moths, are considered an excellent higher eukaryotic model system for their excessive radioresistance amounting up to 100 times higher than human/mammalian cells [1]. Such an exemplary level of radioresistance has been attributed to a variety of factors (summarized in [2]) including lower level of radiation-induced DNA damage, efficient DNA repair, as well as an unusually reduced level of radiation-induced apoptosis [1], [3]. A significantly lower induction of DNA damage in these insect cells may result in very high level of innate radioresistance since DNA damage is considered the most important determinant of radiation-induced cellular lethality [4]. Besides other potential factors such as DNA-protein interactions [2], radiation-induced DNA damage in insect cells could be significantly protected by a more effective antioxidant system [5], that may counter radiation-induced oxidative stress with increased efficiency [6], [7], [8], [9], [10].

Cellular antioxidant mechanisms countering radiation-induced oxidative stress include certain soluble free radical scavenger molecules such as glutathione as well as the enzymatic machinery including superoxide dismutases, catalases and peroxidases. Besides the presence of glutathione at relatively higher intracellular concentrations in Sf9 cells [10], investigations have recently shown an overall enzyme antioxidant capacity that is significantly higher than human cells [5]. The antioxidant role of peroxidases such as thioredoxin peroxidase (TPx) or peroxiredoxin (Prx) has also been known to be quite important since these enzymes offer the first line of defence in the event of oxidative insult by the reactive oxygen species. The TPx protein is known to be actively participating in cellular antioxidant activity in a number of organisms including bacteria, plants and animals [11], [12], [13], and has been reported to function quite efficiently in the insect system [14], [15], [16], [17]. Originally identified as *thiol-specific antioxidant* [18], TPx plays predominant role in the immediate detoxification of free radicals and utilizes thioredoxin as substrate to carry out detoxification of reactive oxygen species (ROS) from the intracellular milieu [19], [20], [21]. The oxidation of thioredoxin (Trx) by the TPx enzyme reduces hydrogen peroxide, peroxynitrites and a wide range of organic hydroperoxides from the intracellular milieu and even provides protection to cells from undergoing stress-induced apoptosis [22], [23]. As a result, TPx might play crucial role in the cellular response to oxidative stress and is reported to be important for regulating cellular radioresistance [24]. Since TPx-mediated oxidative stress response has potentially important role in cellular radioresistance, the present study was conducted to study the nature of TPx and its role in the radioresistance of Sf9 insect cells derived from *Spodoptera frugiperda*, the Fall armyworm (class Insecta; order Lepidoptera).

## Materials and Methods

### Cell Culture

Sf9 cell line, originally derived from ovaries of *Spodoptera frugiperda* (the Fall armyworm; class *Insecta*; order Lepidoptera; family Noctuidae), were maintained in exponential phase as monolayers as described earlier [9]. Axiovert-200 Zeiss inverted DIC microscope (Carl Zeiss, Germany) was used for routinely observations of morphological alterations or cell death. BMG1 cell line derived from human brain malignant glioma tissue was maintained in exponential phase as monolayer, as described earlier [9].

### Cell Morphology Assay for Analysis of Cell Death

For morphological discrimination of apoptotic, necrotic and normal cell population, cells were harvested in uniform suspension in the growth medium and embedded in a thin layer of 0.75% (w/v) low melting point agarose gel over the microscopic slides before fixing with 70% pre-chilled methanol for 10–15 min in the refrigerator. Slides were gently rinsed in distilled water, air-dried at 40°C, and double-stained with 50 µg/ml propidium iodide (Cat number P4170, Sigma USA) along with 10 µg/ml fluorescein isothiocyanate (Cat number F7250, Sigma USA) for 10 min. Morphological observations were made under the fluorescence microscope (Olympus, BX60) and 500 cells per sample were counted manually for calculating the percent apoptotic, necrotic and normal cells based on general morphological features.

### Irradiation and Treatments

For irradiation of Sf9 cells, exponentially growing cultures were used at a dose-rate of 23.33 Gy/min in a ^60^Co gamma chamber (Gamma Chamber 5000, Board of Radiation and Isotope Technology, Department of Atomic Energy, Mumbai, India). All irradiations were carried out at room temperature. In another set of experiments, Etoposide (100 µM, Fresenius Kabi Oncology Ltd. (Phytosid) or Okadaic acid (1 nM and 5 nM, Cat number O8010, Sigma USA) or Roscovitine (5 µM to 25 µM, Cat number R7772, Sigma USA) treatment was given in growth medium for various time intervals.

### Western Blotting

For protein expression analysis Western blotting was performed as described earlier [9] at various time intervals after treatment. For protein oligomerization analysis with non-denaturing-PAGE, Sf9 cells were lysed in non-denaturing conditions without detergent. The lysates were separated by 6% polyacrylamide gel electrophoresis without SDS. The separated proteins from denaturing or non-denaturing gels were transferred to a methanol pre-treated PVDF (Polyvinylidene fluoride) membrane (Pierce). Non-fat dried milk as blocking agent and polyclonal antibodies anti-Prx (H-198, Cat number sc-33574) and anti-Prx (L-20, Cat number sc-23969) from Santacruz Biotech, USA were used for immune-blotting. Immune complexes were detected with appropriate HRP (horseradish peroxidase) conjugated secondary antibody and a chemiluminescence detection system (Pierce, USA). Chemiluminescence signals were captured on X-ray films. Band intensity on X-ray films was measured using ImageQuant software (GE Healthcare). Beta-actin was used as the loading control for normalization.

### Reverse Transcription Polymerase Chain Reaction (RT-PCR)

Total RNA was isolated using RNAeasy kit (Qiagen,USA). Reverse transcription reaction was performed using M-MuLV reverse transcriptase (Fermentas, USA) with oligo-dT^18^ primers and cDNA was taken as template for PCR. Primers (forward primer 5′CCATGGAAATGCCTCTCCAGCTGACC 3′, reverse primer 5′GGATCCCGTTGGCGTCGATGAAGTATTC 3′) were designed to amplify complete Sf-TPx EST sequence yielding ∼0.6 kb of amplicon. PCR reaction was carried out with the following composition - 1X Taq DNA polymerase buffer, 2.5 mMdNTP each, 0.1 µM of each forward and reverse primers, 2 µl cDNA, 2 mM MgCl_2_ and 2 U *Taq* DNA polymerase in 50 µl reaction volume (all from Fermentas). Thermocycler setting was as follows: initial denaturation for 5 min at 95°C, 30 cycles of 1 min at 95°C /1 min at 58°C /1 min at 72°C, final extension for 10 min at 72°C and hold at 4°C until electrophoresis. After PCR, the final products were analysed on 1.5% agarose gel with ethidium bromide staining.

### Intracellular ROS and Nitric Oxide Measurement

5,6-chloromethyl-2′7′-dicholorodihydrofluorescein diacetate (CM-H_2_DCFDA, Cat number D6883, Sigma, USA) at 10 µg/ml concentration was used for measurement of intracellular ROS level while 4-amino-5-methylamino-2′,7′-difluorofluorescein diacetate (DAF-FM diacetate, Cat number D23844, Molecular Probes, USA) 5 µM was used for measurement of intracellular nitric oxide level, as described earlier [9].

### Cell Cycle Analysis

Cell cycle distribution study was performed by analysing the relative DNA content using propidium iodide (50 µg ml^−1^) method in flow-cytometer. The details of this method are described earlier [1].

### Immuno-fluorescence Microscopy

Cells grown on autoclaved cover slips were stained with Mitotracker red (Cat number M7512, Molecular Probes, USA) by mixing Mitotracker Red (100 nM) in growth media and incubating for 30 min at 28°C in dark. Subsequently medium was discarded and cells were washed with wash buffer (PBS with 0.01% bovine serum albumin) and fixed with 2% para-formaldehyde in PBS for overnight at 4°C. These cells were probed with anti-Prx H198 antibody (similar to western blot method) and later with FITC-conjugated secondary antibody using the procedure described earlier [25]. Images were captured using confocal microscope (TCS-SPE; Leica Microsystems) and were processed by Leica LAS AF software.

### siRNA Design and Transfection

EST sequence of Sf-TPx was used for siRNA design using online tool *oligowalk* [26], [27]. The siRNA sequence (5′UAGAAGAACAGUACUACGU3′) obtained from *oligowalk* was custom synthesized commercially and optimized in our lab for specific knock-down. RNAiFect tranfection reagent (Qiagen) was used in 1∶6 ratio for transfection of Sf9 cells according to manufacturer’s recommendations. Knock-down of Sf-TPx protein was assessed by western blotting 24 h after transfection.

### Caspase-3 Activity

Caspase-3 activity was measured by caspase-3 assay kit (Cat number CASP3F, Sigma USA). The caspase-3 fluorimetric assay is based on the hydrolysis of the peptide substrate Ac-DEVD-AMC by caspase-3, resulting in the release of the fluorescent -AMC moiety. Cells were lysed in the lysis buffer provided in the kit and the assay for active caspase3 was performed according the manufacturer’s recommendation. The mean fluorescence values of triplicates were plotted to determine variation in caspase-3 activation.

### *In-silico* Analysis

*S. frugiperda*TPx sequences [28] were used for sequence similarity study with other known sequences by use of ClustalW available in BioEdit software. Intracellular localization prediction for all the sequences was done by using online tool WolfPsort [29] or MitoProt [30].

### Statistical Analysis

For the analysis of band intensity in western blots or of fluorescence values in the caspase-3 assay, the *t*-test was used with *p≤*0.05 as significance level. SEM ± was used in Microsoft Excel for bar graph preparations. Standard error was calculated and displayed on the bar graph as ± error bars.

## Results

### *In silico* Characterization and Detection of Thioredoxin Peroxidase in Sf9 Cells

**Figure 1:**
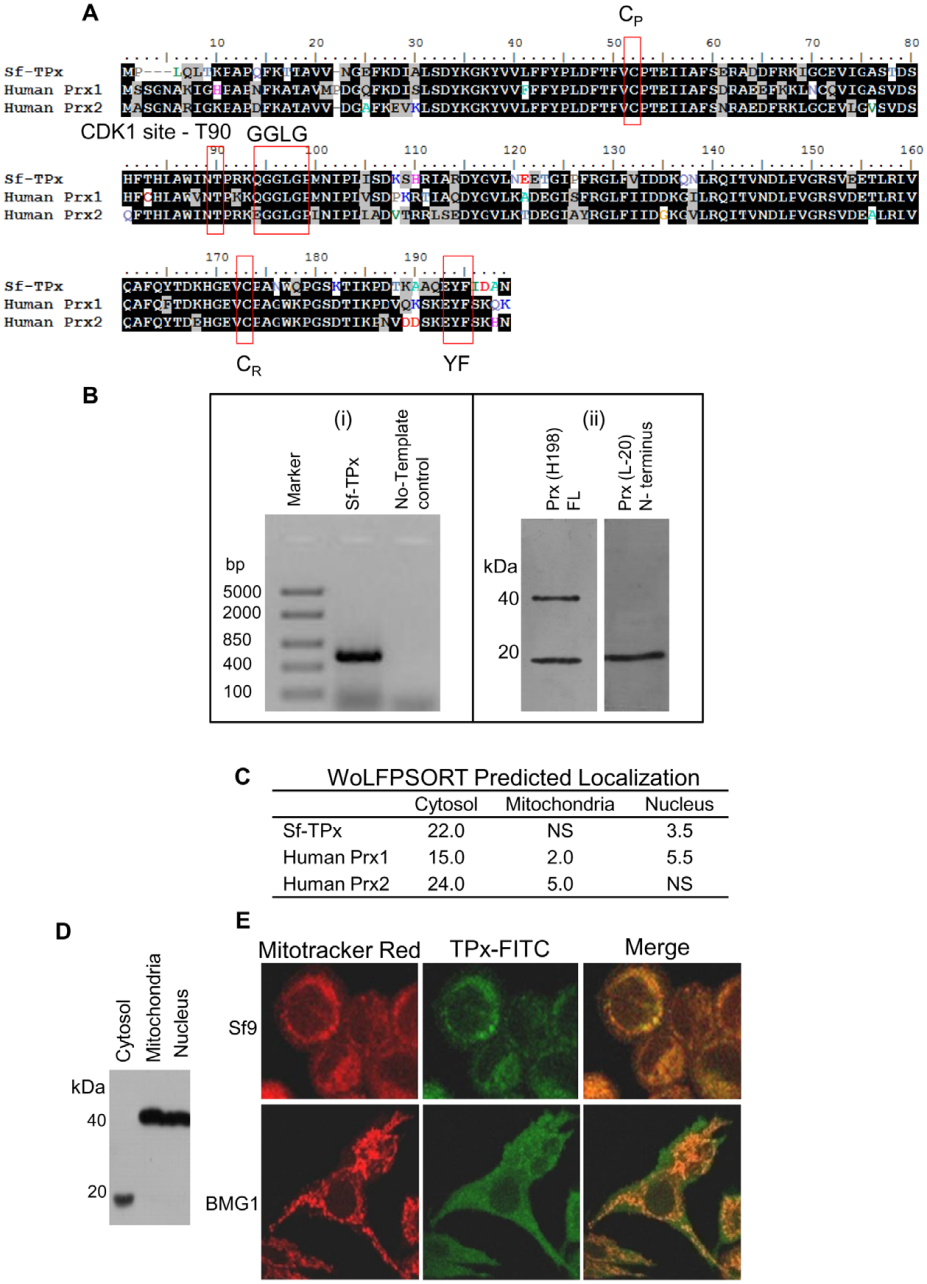
*In silico* and experimental evidence for the presence and intracellular localization of thioredoxin peroxidase in *Spodoptera frugiperda* cells. (A) Sequence alignment of Sf-TPx with human Prx1 and Prx2 shows high sequence homology and conservation of GGLG and YF motifs and active site cysteine residues (C_P_ and C_R_) between the species. Sf-TPx1 - Butterflybase Acc. No.SFP00903_3; Prx1 - NCBI Acc. No.NM_002574; Prx2 - NCBI Acc. No. NM_005809. (B) Reverse transcription PCR of Sf9 total RNA shows amplification band of ∼600 bp (i); Western blot of total proteins by human Prx full length specific antibody (Prx H198) shows interaction with two bands (∼20 kDa and ∼40 kDa) while the human Prx N-terminus specific antibody (Prx L-20) shows interaction with only a single 20 kDa band (ii). (C)*In-silico* analysis (WoLFPSORT) shows that Sf-TPx is present predominantly in cytosol which is similar to that of human Prx’s localization. (D) Western blot of cytosolic and mitochondrial fractions of Sf9 cellular proteins interestingly shows that 20 kDa band is present in cytosolic fraction whereas 40 kDa band is in the mitochondrial fraction. (E) Confocal microscopy images using mitochondria-specific dye Mitotracker Red along with anti-hPrx FL-FITC antibody also confirms higher proportion of Sf-TPx’s with mitochondrial localization as compared to the human Prx. BMG1 cells derived from human brain malignant glioma were used for comparison of intracellular localization of TPx with Sf9 cells.

Analysis of EST database has previously revealed the *Spodoptera frugiperda* thioredoxin peroxidases (Sf-TPx’s) [28]. These orthologues show high sequence similarity and domain conservation with human Prx1 and Prx2 (hPrx1, hPrx2) (Fig. 1A), including the two highly important and conserved cysteine residues, peroxidatic cysteine (C_P_) and reducing cysteine (C_R_), thus suggesting that Sf-TPx is a member of *typical 2-Cys* class of thioredoxin peroxidases. The C_P_ of hPrx1 (Cys52) corresponds to Cys48 and C_R_ (Cys173) corresponds to Cys169 Sf-TPx. The GGLG (91–94 in Sf-TPx) and YF (190–91 Sf-TPx) motifs, important for the regulation of peroxidase activity, were also found conserved in the TPx of *S. frugiperda*. A threonine residue (T90 human Prx1 numbering) which is a site for CDK1 mediated phosphorylation in Prx1 was also found conserved in Sf-TPx, indicating possible involvement of cell cycle check point regulators in regulation of Sf-TPx activity in Sf9 cells (Fig. 1A).

PCR amplification from the cDNA prepared from Sf9 RNA using primers based on EST sequence of Sf-TPx yielded a band of approximately 0.6 kb which corresponds to the 585 bp long sequence of Sf-TPx EST (Fig. 1B-i). Anti-human Prx (FL and N-terminus) polyclonal antibodies yielded specific bands of approximately 20 kDa (Sf-TPx) from Sf9 cell protein extracts in western immunoblots which corresponds to the theoretically calculated molecular mass of Sf-TPx as well as known molecular mass of human Prx1 and Prx2 (Fig. 1B-ii). We repeatedly observed another specific band of around 40 kDa in immunoblots using Prx FL antibody (Fig 1B-ii), which may be because of presence of other isoforms of Sf-TPx, although no EST sequence of TPx corresponding to 40 kDa size could be identified.

Intracellular location of thioredoxin peroxidase is an important factor for effective detoxification of stress-induced ROS [31], and WoLFPSORT(*In-silico*) analysis suggests that Sf-TPx may be present in the cytosol of *S. frugiperda* cells (Fig. 1C). Using the Western immunoblotting with an antibody (Prx-H198) against full length TPx, we observed a 20 kDa protein band in the cytosolic fraction of Sf9 cells (Fig. 1D), confirming that a cytosolic isoform of TPx exists in Sf9 cells as indicated from *in silico* analysis. The 40 kDa band observed above was apparently present in mitochondrial as well as nuclear fraction and is being investigated further. Confocal microscopy of Sf9 and human glioma cells (BMG-1) with the mitochondria specific dye mitotracker red and anti-human PrxFL antibody (FITC) confirmed cytosolic localization of TPx’s in both insect Sf9 and human BMG-1 cells (Fig. 1E).

### Sf-TPx is Significantly Upregulated by Sub-lethal Dose of γ-radiation

**Figure 2:**
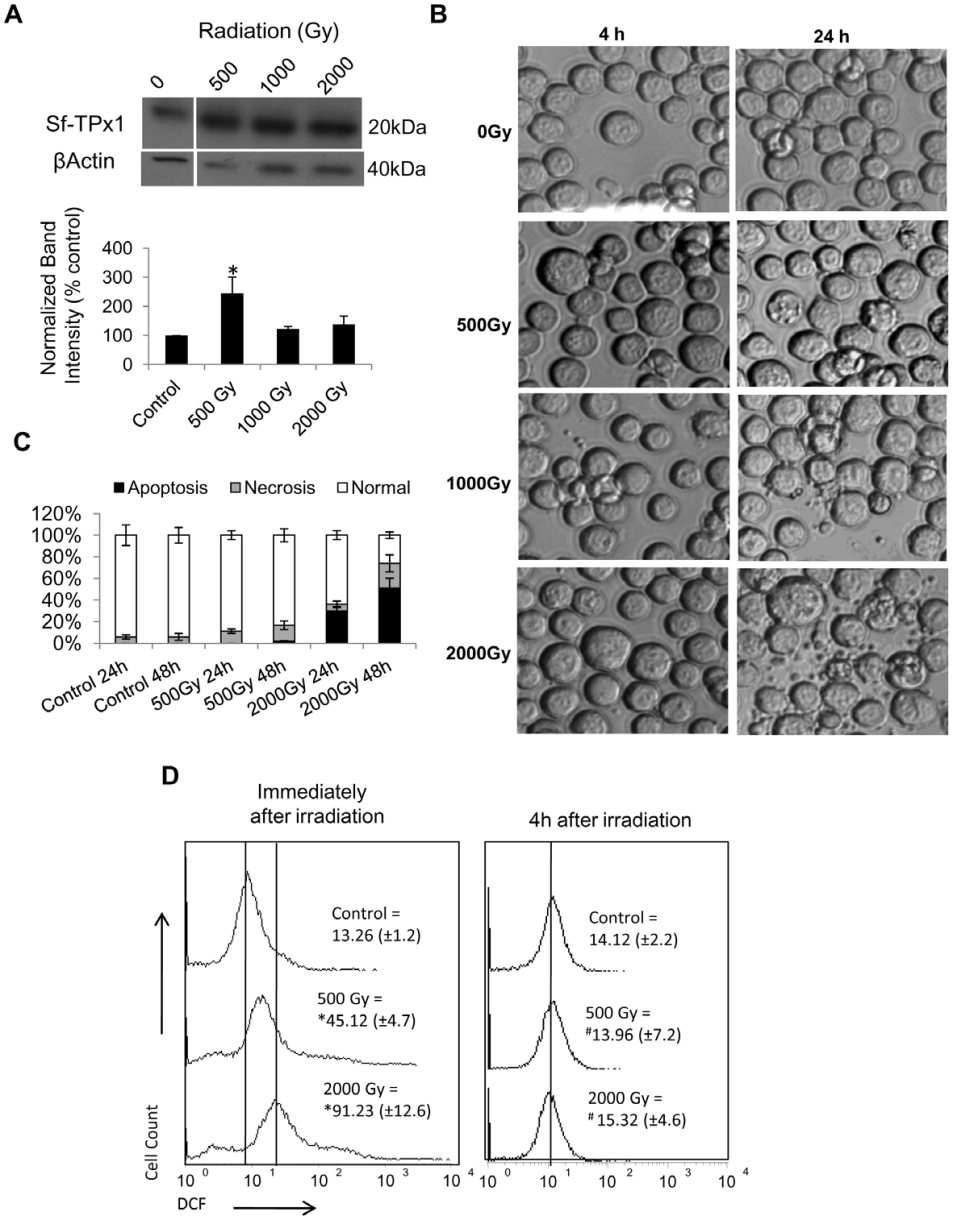
Sf9 thioredoxin peroxidase is significantly upregulated by irradiation at sub-lethal dose of 500 Gy and not at higher lethal doses. (A) Sf-TPx1 western blot shows increase in expression at 4 h post-irradiation when probed with anti-hTPx (PrxH198) antibody. Bar graph represents mean band intensity of three independent experiments after normalization with β-Actin expression using densitometric analysis. (‘*’ indicates *p≤*0.05 with respect to untreated control). (B) Nomarski-DIC images showing dose-dependent induction of apoptosis in 1000 Gy–2000 Gy irradiated Sf9 cells at 24 h post-irradiation. (C) Intracellular ROS levels measured by flow cytometry of live cells stained with H_2_DCF-DA show dose-dependent increase immediately following irradiation that was reverted to normal level as early as 4 h post-irradiation. Data are presented as frequency histograms of DCF fluorescence representing one of the three independent experiments that yielded closely comparable results and mean fluorescence intensities are shown in the panel. Variation in mean value is shown within parenthesis. (*p≤0.05; ^#^ *p*≥0.1) (D) Apoptotic, necrotic and normal cells counted by PI/FITC staining of agarose-embedded and fixed cells (24 h and 48 h post-irradiation) clearly shows dose-dependent increase in apoptosis following irradiation at 500 Gy–2000 Gy, with highly significant induction at 2000 Gy. Data represent mean of three experiments with error bars showing standard error.

Radiation-induced ROS and RNS may elicit increase in expression and/or activity of antioxidant components. Alterations in the expression of Sf-TPx were thus assessed following radiation-induced oxidative stress at high doses (500 Gy-2000 Gy). While Sf-TPx was found to be significantly upregulated (Fig. 2A) following irradiation at 500 Gy dose at which these cells easily survive, no increase in its expression could be observed following irradiation at higher doses (1000 Gy and 2000 Gy) that caused significant apoptotic cell death by 72 h in a dose-dependent manner (Fig. 2B, Fig. 2C).

Further, radiation-induced intracellular ROS/RNS was also investigated using the H_2_DCFDA dye which is sensitive to oxidation by hydrogen peroxide (H_2_O_2_), hydroxyl radical (OH^•^) as well as peroxynitrite (ONOO^−^). We observed a dose-dependent significant increase in DCF fluorescence when measured immediately after irradiation (Fig. 2D), which interestingly returned to normal levels within 4 h at all doses, indicating a strong antioxidant system in Sf9 cells as discussed later.

### Activation of Radiation-induced G2/M Checkpoint in Sf9 Cells is Associated with Oligomeric State Transition of TPx

**Figure 3:**
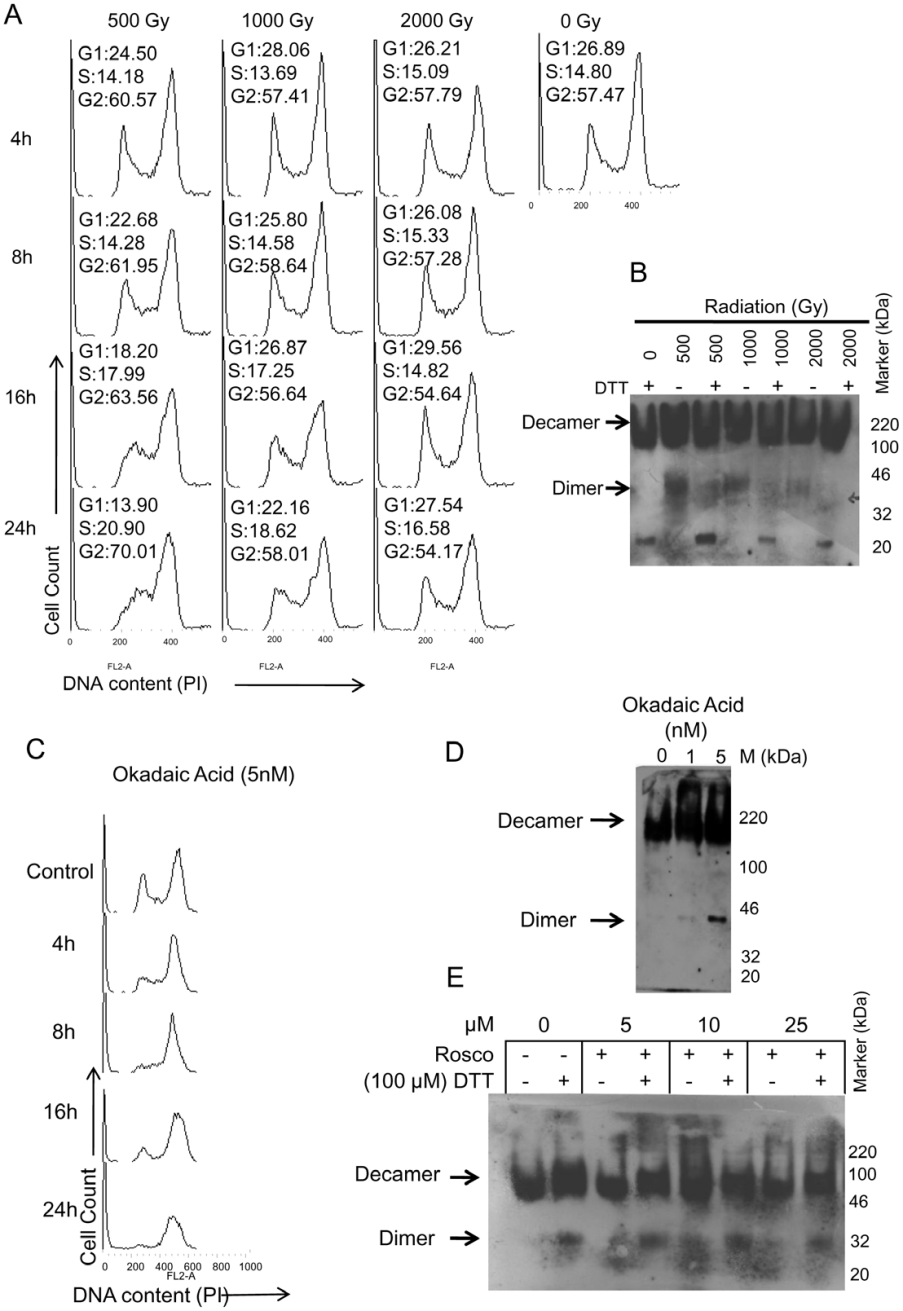
Cell cycle progression delay at G2/M phase is associated with the accumulation of dimer/oligomer form of Sf-TPx. (A) DNA content flow histograms depicting radiation-induced cell cycle delay in Sf9 cells irradiated at 500 Gy display prominent G2/M block, while the higher doses 1000 Gy and 2000 Gy caused checkpoints in other phases and hence yielded relatively lower G2/M sub-population compared to 500 Gy. Percentage of gated population in G1, S and G2 phases are shown in panels. (B) Native-PAGE western immunoblot showing maximum accumulation of Sf-TPx dimers following 500 Gy dose that corresponds with the extent of G2/M block. (C) DNA flow histogram of Sf9 cells treated with okadaic acid showing cell cycle delay at G2/M phase. (D) Native PAGE western blot of proteins extracted from the okadaic acid treated Sf9 cells showing accumulation of oligomeric form of Sf-TPx. (E) Native PAGE western blot of proteins extracted from the Sf9 cells treated with CDK inhibitor roscovitine shows accumulation of oligomeric form of Sf-TPx in Sf9 cells.

Using the flow cytometric analysis of propidium iodide stained cells, we observed the induction of cell cycle checkpoint at G2/M phase at 8 h to 24 h following 500 Gy irradiation (Fig. 3A), whereas cells remained arrested in all phases of cell cycle at the higher radiation doses tested, viz., 1000 Gy–2000 Gy. Since CDKs are known to phosphorylate TPx’s at a certain residue and favour the formation of decamer form of TPx (∼200 kDa), any disturbance in the CDK activity by oxidative stress could slow down this process and may inhibit TPx decamer formation. In such condition, prevalence of dimers may favour an increased peroxidative activity. Therefore, we assessed oligomerization status of Sf-TPx’s in the irradiated Sf9 cells by using non-denaturing PAGE western immunoblot of lysates from treated cells in the presence or absence of dithiothretol (DTT). DTT is a strong reducing agent and is able to reduce Disulfide Bridges, thus yielding monomers from oligomeric forms of proteins. We observed the presence of dimers in the irradiated samples, which further dissociated into monomers in the presence of DTT (Fig. 3B). This observation indicates the dissociation of 200 kDa decamer forms into the dimers of TPxs, and strongly indicates that TPx oligomerization may be associated with the induction of G2/M cell cycle checkpoint in the high-dose irradiated Sf9 cells.

We further used okadaic acid, a phosphatase inhibitor that is known to induce cell cycle perturbation. Induction of cell cycle delay was observed at G2/M phase (Fig. 3C), which was associated with significant accumulation of Sf-TPx dimeric form (Fig. 3D) detected by non-denaturing PAGE western blot analysis. Since CDC2 is known to regulate G2/M cell cycle transition and also phosphorylates TPx which leads to its decamer formation [32], we used Roscovitine, an inhibitor of CDKs (5 µM to 25 µM), to determine its effect on decamer to dimer from interchange of Sf-TPx in the Sf9 cells. Significant accumulation of dimer form of Sf-TPx (Fig. 3E) was evident following roscovitine treatment, which confirms that Sf9 cell cycle transition block is associated with an active transition of the oligomeric state of TPx.

### Knock-down of Sf-TPx Enhances Radiation-induced Oxidative Stress and Apoptosis, but Fails to Induce Nitrosative Stress

**Figure 4:**
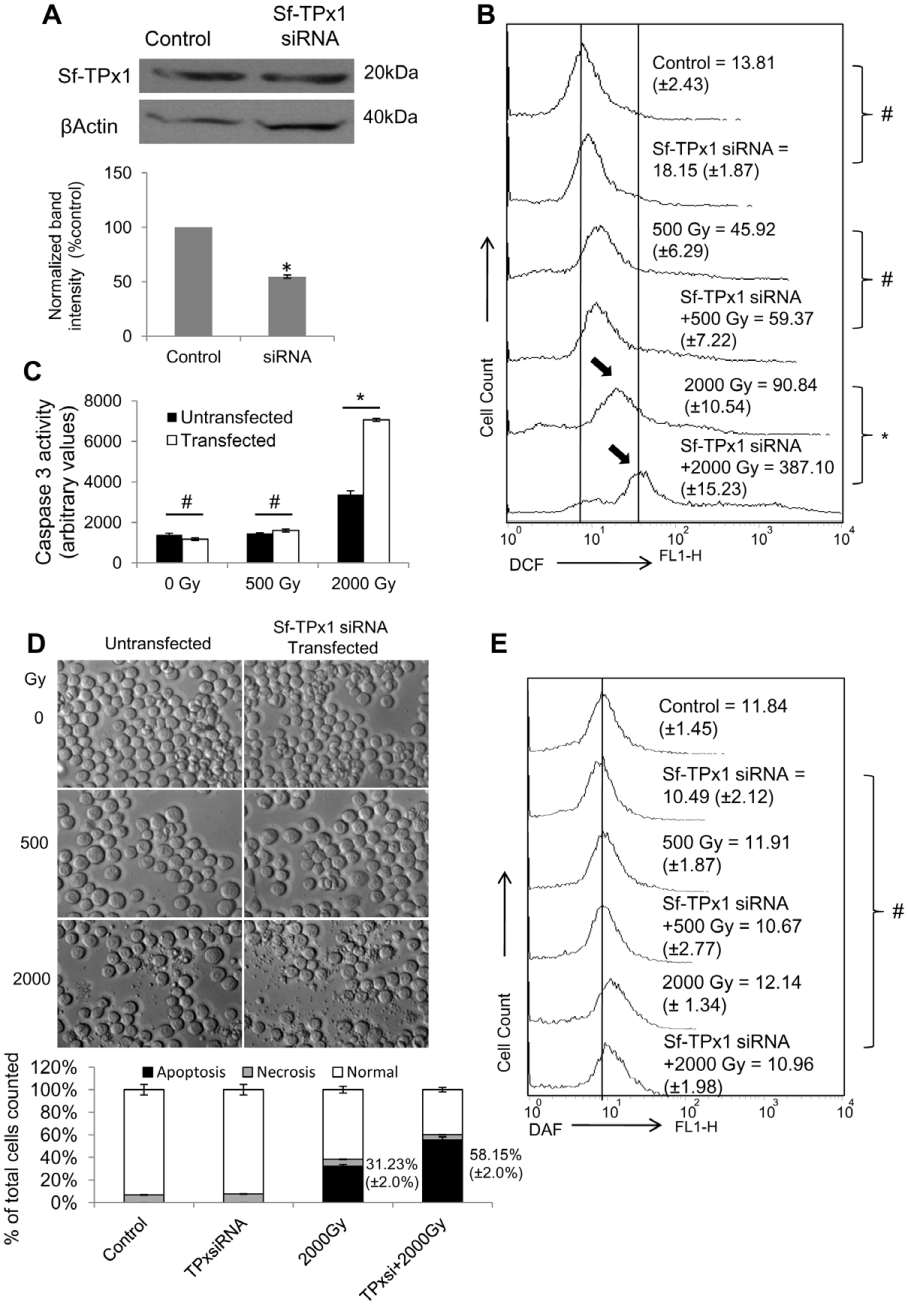
Sf-TPx1 knock-down caused significant increase in radiation-induced ROS generation and cell death. Sf9 cells were transfected with 1 µg siRNA against Sf-TPx1 with 6 µl RNAiFect transfection reagent and incubated for 24 h at 28°C. (A) Western immunoblot shows significant decrease in Sf-TPx1 level 24 h after siRNA transfection. Bar graph showing mean normalized band intensity of three independent experiments and ‘*’ indicates *p≤*0.05 with respect to untransfected control. (B) Flow histogram shows increased induction of radiation-induced intracellular ROS in the TPx-knockdown Sf9 cells. The levels of ROS were assessed by incubating transfected and untranfected Sf9 cells with H_2_DCFDA 30 min prior to irradiation and DCF flouroscence was measured immediately after irradiation. Mean fluorescence intensities are shown in panel and variation from mean is shown within parenthesis. (*p≤0.05; ^#^ *p*≥0.1). (C) Bar graph representing mean capsase-3 activity in Sf9 cells 24 h post-irradiation, from three independent experiments. Caspase-3 activity was significantly induced by radiation in TPx-knockdown Sf9 cells. ‘*’ indicates *p≤*0.05 and ‘#’ indicates *p*≥0.1 (D) DIC microscopy images and PI/FITC staining based determination of percentage of apoptotic and necrotic cell population (bar graph) shows approximately two-fold increase in apoptosis in the 2000 Gy-irradiated cells following TPx knockdown. (E) Flow histogram showing unaltered level of intracellular NO, assessed using DAF-2DA staining of transfected and untranfected Sf9 cells. DAF flouroscence was measured immediately after irradiation, and mean fluorescence intensities are shown. The variation from mean fluorescence intensity is shown within parenthesis (^#^ values not significantly different from control).

Further we assessed the radiation-induced ROS induction as well as cell death when the functioning of thioredoxin system was compromised through RNA interference. We designed siRNA by utilizing EST sequence database and knocked down Sf-TPx prior to irradiation (Fig. 4A). Sf-TPx knock-down resulted in significantly higher level of ROS generation following 2000 Gy (Fig. 4B) associated with higher caspase-3 activity (Fig. 4C)and approximately two-fold induction of apoptotic cell death (Fig. 4D).

Since TPx is known to be up-regulated by increased levels of nitric oxide (NO) and contributes in regulating nitrosative stress [33], [34], [35], we further assessed the level of radiation-induced NO level in the TPx-knockdown Sf9 cells using NO specific dye DAF-2DA. Sf-TPx knock-down did not result in any increase in the radiation-induced NO generation (Fig. 4E).

### Etoposide-induced Sf9 Cell Death Lacking High ROS Generation Remained Unaffected by Sf-TPx Knock-down

**Figure 5:**
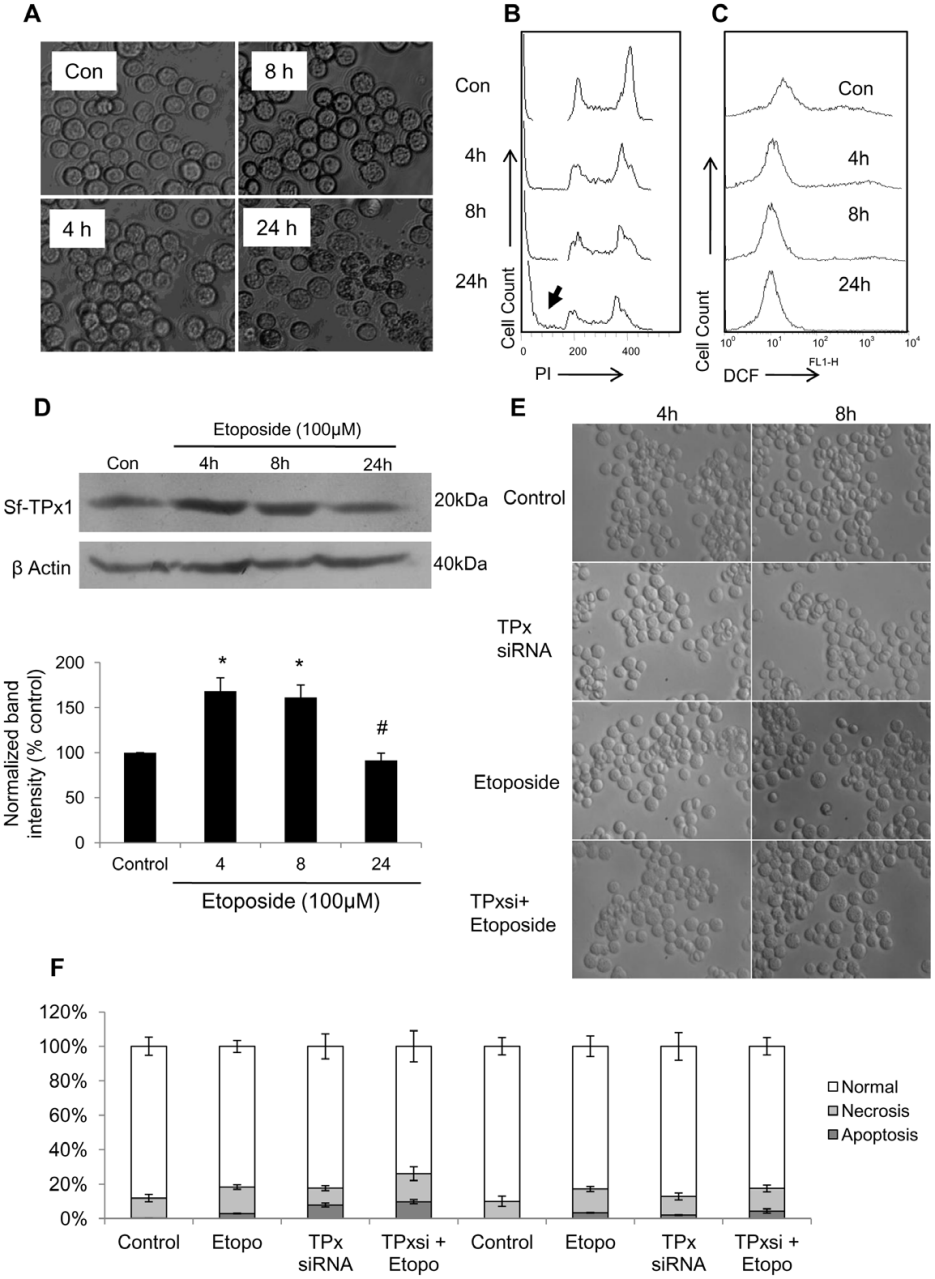
Lack of increase in the etoposide-induced cell death following Sf-TPx knockdown. Sf9 cells were treated with 100 µM Etoposide for 4 h, 8 h, and 24 h. (A) DIC microscopy images of etoposide-treated cells showing death induction following treatment. (B) Flow histograms of propidium iodide stained etoposide-treated Sf9 cells showing cell death due to etoposide treatment. (C) Flow histograms of DCF fluorescence from etoposide treated Sf9 cells show no significant alteration (*p*≥0.1) in ROS level by etoposide. (D) Sf-TPx1 expression was moderately up-regulated following etoposide treatment. Bar graph of normalized band intensity of western blot showing mean of three independent experiments.* indicates *p≤*0.05 and # indicates *p*≥0.1 with respect to untreated control. (E) DIC microscopy images show lack of alteration in the etoposide-induced cell death in cells following Sf-TPx1 knock-down. (F) Morphological analysis of apoptotic, necrotic and normal cells using PI/FITC staining of agarose-embedded fixed cells confirm lack of alteration in etoposide-induced cell death following Sf-TPx knock-down. Data represent mean of three experiments with error bars showing standard error.

Etoposide, a topoisomerase inhibitor, causes DNA damage and cell death without inducing significant generation of ROS/RNS. In order to further demonstrate that TPx is primarily involved in the oxidative stress induced damage in Sf9 cells, we assessed its role in etoposide induced cell death in Sf9 cells. DIC microscopy (Fig. 5A) and DNA content analysis by flowcytometry (Fig. 5B) show increased cell death by etoposide. However, the intracellular ROS levels failed to increase with time during etoposide treatment (Fig. 5C). Sf-TPx was found to be up-regulated by etoposide treatment (Fig. 5D) implying possible involvement of this enzyme in response to etoposide treatment. However, knocking down of Sf-TPx did not cause any significant increase in the level of cell death (Fig. 5E, Fig. 5F), hence confirming that TPx may not play major role in ROS-independent cell death.

## Discussion

The present study importantly demonstrates that thioredoxin peroxidase, a potentially important component of the antioxidant defence, plays a noticeable role in the radioresistance of Lepidopteran Sf9 insect cells. Firstly, we characterized thioredoxin peroxidase in Sf9 cells using bioinformatics and other approaches. The *in silico* sequence homology and domain conservation of *Spodoptera* TPx from EST database with the human system as well as western blotting using anti-human Prx antibody (Fig. 1A, 1B) indicated a possible good conservation between the human and insect TPx orthologues [23], [36]. The predicted cytosolic localization of Sf-TPx similar to human Prx1 and Prx2 was experimentally confirmed using confocal microscopy and western blot of cytosolic, nuclear and mitochondrial fractions, and strongly suggests that thioredoxin peroxidase may be efficiently functioning in these insect cells under conditions of oxidative stress.

A significant increase observed in the expression of Sf-TPx following irradiation at 500 Gy (Fig. 2A) was associated with a negligible radiation-induced ROS generation and subdued cell death response (Fig. 2B, 2C). In contrast, higher doses (1000 Gy–2000 Gy) failed to upregulate TPx (Fig. 2A) and caused significant ROS generation (Fig. 2C) as well as apoptotic cell death by 72 h post-irradiation (Fig. 2D). Therefore, upregulated expression of TPx enzyme seems to coincide with the resistant response of cells against radiation-induced ROS and cell death. Higher expression of certain isoforms of TPx as observed in several cancers [37], [38], [39], [40], [41], [42] has been shown to inhibit stress-induced increase in intracellular H_2_O_2_ [43] and is also associated with resistance to radiation [24] or chemical stress [44]. Present findings in these model radioresistant cells further support the putative role of TPx in cellular radioresistance.

Various types of TPx homologues/orthologues existing in different organisms are divided into three categories based on their active site cysteine residues, viz., the *typical 2-Cys TPx*, the *atypical 2-Cys TPx*, and the *1-Cys TPx*. The typical *2-Cys-TPx* is characterized by the presence of two cysteine residues on different monomers of the dimeric protein at the active site. The regulation of activity of typical *2-Cys TPx* importantly involves dimer (40–44 kDa) to decamer (200 kDa) transition [31], [45]. The decamer conformation is made by integration of five dimer units in a doughnut shaped arrangement [31] and mainly carries out chaperonic activity [45]. The dissociation of decamer into dimer is required for peroxidase activity and is regulated by several factors including CDK1 mediated phosphorylation [32], [46]. The CDK1 mediated phosphorylation of Thr90 of hTPx1 leads to decamer formation causing significant reduction in peroxidase activity, while CDK1 inhibition by roscovitine has been reported to result in accumulation of dimer form leading to higher peroxidase activity of hPrx1 [32]. Therefore, the transition of TPx oligomeric state in response to any stress may play important role in the overall stress resistance/sensitivity of cells. In the present study, interestingly we observed an accumulation of the dimer form of Sf-TPx’s in the 500 Gy-irradiated Sf9 cells using non-denaturing PAGE gel, which was associated with cell cycle delay at G2/M phase (Fig. 3A, 3B). In order to further confirm the possible association of TPx dimer formation with stress-mediated G2/M transition block, we further treated Sf9 cells with okadaic acid (OA), a phosphatase inhibitor, as well as with a specific CDK1 inhibitor roscovitine [47], [48], and found dimer form accumulation in both the cases (Fig. 3D, 3E). The conservation of CDK1 phosphorylation site in Sf-TPx and accumulation of dimer forms following CDK1 inhibition by roscovitine strongly indicate that CDK1 inhibition associated with radiation/stress induced G2/M delay could be associated with oligomeric state transition of TPx in Sf9 cell, which is important for enhancing its peroxidase activity that may in turn contribute in cellular stress tolerance.

Abrogation of thioredoxin system by TPx knock-down causes increased radiation-induced lethality in several mammalian cell types [24], [49], [50]. For confirming the role of TPx in the radioresistant response of Sf9 insect cells, we conducted transient knock-down of TPx using the RNA interference approach. A partial (∼50%) knock-down of Sf-TPx indeed resulted in a significant increase in the radiation-induced ROS generation as well as apoptosis mediated by caspase-3 activation (Fig. 4C). The effect was quite prominent at 2000 Gy, enhancing caspase activity and apoptotic cell death by as much as two-fold (Fig. 4C, 4D). These results thus importantly show that TPx indeed plays an important role in the radiation resistance of Sf9 Lepidopteran cells. However, this effect was prominently visible at highest doses tested which may be due to additional antioxidant/other factors also playing equally significant role.

In order to further test whether TPx plays any significant role in the cell death induced when there is no strong generation of oxidative stress, we treated Sf9 cells with Etoposide that causes covalent linkage between DNA and topoisomerase-II to stabilize the cleavable complex and leads to DNA damage and cell death [51], [52]. Although etoposide treatment has been shown to induce translocation of Sf-p53 into nucleus indicating induction of DNA damage [53], no alteration in the intracellular ROS/RNS levels could be observed in the present study (Fig. 4C) even though it was associated with an increase in Sf-TPx expression level. Moreover, Sf-TPx knock-down failed to cause any significant increase in the etoposide-induced cell death (Fig. 5E, 5F), which indicates an insignificant role of Sf-TPx in stress events that may not involve extensive oxidative damage.

Recently we have reported that NOS activity is negligible in Sf9 cells in comparison to human cells, even very high doses of γ-radiation up to 2000 Gy failing to induce NOS-mediated nitrosative stress in these insect cells [9]. NOS activity and NO levels were also observed to be refractory to radiation-induced cytosolic release of calcium from intracellular reservoirs. Since TPx is also known to protect human cells from nitrosative stress and reactive nitrogenous intermediates [35], [54], and its expression reportedly increases during the nitrosative stress [33], [34], we checked whether alteration in the levels of TPx could be associated with any change in intracellular NO generation. Quite importantly, no alteration could be detected in the NO levels after Sf-TPx knock-down in the high-dose irradiated cells (Fig. 4E). This data confirms our recent findings [9] that radiation-induced cell death in Sf9 cells is primarily mediated by ROS with negligible contribution from nitric oxide or other reactive nitrogen intermediates.

### Conclusions

In summary, our study strongly indicates the existence of thioredoxin peroxidase and also demonstrates its involvement in the radiation response of Lepidopteran (Sf9) insect cells. Stress-induced G2/M cell cycle checkpoint in Sf9 cells was associated with the oligomeric state transition of Sf-TPx (dimer formation) which is known to favour the latter’s peroxidase activity [32] and suggests an active role of TPx in the Sf9 radiation/stress response. Finally, a highly significant increase in the high dose radiation-induced apoptosis following knock-down of Sf-TPx clearly demonstrates an important role of TPx in the Lepidopteran radiation resistance. This is the first study presenting evidence for thioredoxin peroxidase involved in the extreme radioresistance displayed by Lepidopteran insect cells, and hence provides an important insight into these cells’ antioxidant mechanisms.
